# Low Preoperative Free Triiodothyronine, Particularly in Females, Is Associated With Reduced Weight Loss and Diabetes Remission After Bariatric Surgery in Euthyroid Adults

**DOI:** 10.1002/dmrr.70228

**Published:** 2026-09-14

**Authors:** Shaobo Li, Yinfang Tu, Hongwei Zhang, Yunfeng Xiao, Yuqian Bao, Jun Yin, Yujin Ma, Haoyong Yu, Fengjing Liu

**Affiliations:** ^1^ Department of Endocrinology and Metabolism Shanghai Sixth People’s Hospital Affiliated to Shanghai Jiao Tong University School of Medicine Shanghai Clinical Center for Diabetes Shanghai Key Clinical Center for Metabolic Disease, Shanghai Diabetes Institute Shanghai Key Laboratory of Diabetes Mellitus Shanghai China; ^2^ Henan Key Laboratory of Rare Diseases Endocrinology and Metabolism Center The First Affiliated Hospital, and College of Clinical Medicine of Henan University of Science and Technology Luoyang China; ^3^ Department of General Surgery Shanghai Sixth People’s Hospital Affiliated to Shanghai Jiao Tong University School of Medicine Shanghai China; ^4^ Department of Radiology Shanghai Sixth People’s Hospital Affiliated to Shanghai Jiao Tong University School of Medicine Shanghai China

**Keywords:** bariatric surgery, diabetes remission, obesity, thyroid function, weight loss

## Abstract

**Aims:**

With the rising prominence of GLP‐1 receptor agonists, identifying predictors of optimal bariatric surgery candidates has become increasingly important. We investigated cross‐sectional relationships between thyroid hormones and obesity‐related parameters, and evaluated the predictive value of baseline thyroid hormones for weight loss (WL) and diabetes remission (DR) after bariatric surgery.

**Methods:**

This study included a cross‐sectional cohort of 1567 euthyroid patients with overweight or obesity, of whom 1034 with complete 1 year postoperative data were included in the longitudinal analysis. Body composition was assessed using validated predictive equations and abdominal MRI. The primary outcomes were successful WL (total WL ≥ 25%) and DR (fasting glucose < 5.6 mmol/L and HbA1c < 6.0% without antidiabetic medications) during the 3 year follow‐up.

**Results:**

Breakpoint analysis indicated that free triiodothyronine (FT3) plateaued beyond an age threshold while showing a linear correlation with fat‐free mass index. Higher baseline FT3 tertiles were significantly linked to increased likelihood of achieving successful WL and DR at 1 year follow‐up, with stronger associations in females. Optimal FT3 thresholds were identified as 4.75 (females) and 5.05 (males) for WL, and 4.57 (females) and 5.00 (males) for DR. Preoperative FT3 levels below these thresholds were significantly associated with reduced WL and DR over 3 years.

**Conclusions:**

Serum FT3 exhibits an age‐related breakpoint effect, and low preoperative FT3, particularly in females, is a critical predictor of suboptimal short‐ and long‐term WL and DR success post‐bariatric surgery. Sex‐specific FT3 thresholds may aid in preoperative risk stratification and inform personalised treatment decisions in the era of expanding obesity therapeutics.

## 1 Introduction

Type 2 diabetes mellitus (T2DM) and obesity represent major global health concerns [1, 2]. Although GLP‐1 receptor agonists have revolutionised obesity management, bariatric surgery remains the most durable intervention for severe obesity and T2DM [3]. However, predicting postoperative weight loss (WL) and diabetes remission (DR) remains challenging, as 20%–30% of patients experience inadequate weight reduction and suboptimal glycaemic control [4, 5]. Moreover, even when patients initially achieve successful WL and DR, these issues can recur [6, 7]. Identifying the baseline determinants of WL and DR is crucial for enhancing the effectiveness of bariatric surgery and reducing the risk of relapse.

Obesity is an energy imbalance disorder, with weight loss primarily depending on the extent of postoperative negative energy balance, in which thyroid hormones (TH) play a key role in regulating resting energy expenditure (REE) [8, 9, 10]. Hypothyroidism is associated with hypometabolism and weight gain, while hyperthyroidism to weight loss [10]. Low circulating levels of TH, even within normal reference concentrations, can be associated with the tendency to gain weight [11]. Free triiodothyronine (FT3) is a crucial component of TH, with low levels observed in fasting and underweight individuals, and elevated levels found in overfeeding and obesity [12, 13], suggesting that FT3 plays a central role in weight regulation and nutrient sensing. However, the impact of baseline TH levels, especially FT3, on WL after bariatric surgery remains largely unknown.

Glycaemic benefits from bariatric surgery are often attributed to sustained weight reduction [14, 15]. Hypothyroidism may lead to reduced REE and weight gain, potentially facilitating the incidence of T2DM [16, 17]. Meanwhile, thyroid dysfunction increases insulin resistance in muscle and adipose tissue, and decreases glucose transport in myocytes [18]. Improved insulin sensitivity is a key mechanism for DR following bariatric surgery. Evidence has suggested that low circulating levels of TH, even within laboratory reference ranges, may be related to an elevated risk of developing T2DM, especially among individuals with prediabetes [16, 19, 20]. Therefore, it is reasonable to deduce that baseline TH, particularly FT3, may influence glycaemic homoeostasis after bariatric surgery. However, no longitudinal studies have yet explored the associations of baseline TH levels with DR after bariatric surgery.

Considering these factors, we explored the cross‐sectional relationships of thyroid function with adiposity and metabolic parameters in patients with overweight or obesity, and longitudinally investigated the associations of baseline thyroid hormones with short‐ and long‐term WL and DR after bariatric surgery.

## 2 Materials and Methods

### 2.1 Study Design and Population

The data presented are from a prospective cohort study on bariatric surgery (Chinese Clinical Trial Registration number: ChiCTR1900028513; https://www.chictr.org.cn/). From February 2011 to November 2022, a total of 1810 patients were assessed for eligibility, with 1567 enroled in the cross‐sectional study. We conducted a longitudinal study on weight loss outcomes among 1034 patients who underwent bariatric surgery and had complete 1‐year follow‐up data, and further investigated diabetes remission in patients diagnosed with T2DM within this cohort. A detailed flowchart is presented in Supporting Information S1: Figure S1. All patients met the guidelines for bariatric surgery [21]. Inclusion criteria were as follows: age, 18–67 years; BMI, 25.0–55.0 kg/m^2^; and fasting C‐peptide level, > 1.0 ng/mL. Exclusion criteria included history of thyroid disease, treatment with thyroid hormone or antithyroid drugs, preoperative thyroid assessment consistent with overt hypothyroidism (elevated thyroid‐stimulating hormone (TSH) with low free thyroxine (FT4)) or overt hyperthyroidism (low TSH with high FT4 and/or high FT3), missing preoperative TSH, FT4 or FT3 data, anaemia, latent autoimmune diabetes, psychiatric or eating disorders, a history of gastrointestinal or malignant diseases, and previous gastrointestinal surgery. This study was conducted in accordance with the Declaration of Helsinki and was approved by the Ethics Committee of Shanghai Jiao Tong University School of Medicine Affiliated Sixth People's Hospital on 24 December 2019 (approval number 2019‐KY‐049(K)). Informed consent was obtained from each patient.

### 2.2 Clinical and Biochemical Measurements

The data collection process was previously described [22]. In brief, anthropometric characteristics, serum biochemical parameters, and medication history at baseline and postoperative follow‐up were collected by trained investigators. As part of the inpatient study protocol, routine abdominal magnetic resonance imaging (MRI) was conducted preoperatively and during postoperative follow‐up. Patients were asked to complete an inpatient or outpatient evaluation 6 months after surgery and annually thereafter. Patients lost to follow up due to various factors (e.g., work commitments, geographical distance or changes in contact information) were contacted multiple times by telephone or mail to minimise attrition. Additionally, FT3, FT4, and TSH were measured by electrochemiluminescence immunoassays on the Cobas e601 analyser (Roche Diagnostics GmbH, Mannheim, Germany), with intra‐assay coefficients of variation < 7.0%, < 5.0%, and < 3.0% for FT3, FT4, and TSH, respectively, and inter‐assay coefficients of variation < 8.0%, < 7.0%, and < 8.0%, respectively.

### 2.3 Measurement of Body Composition

Body fat percentage (BFP), fat‐free mass index (FFMI), and fat mass index (FMI) were measured using a gold‐standard‐derived predictive equation. Abdominal MRI scans were performed at the third lumbar vertebra, and SliceOmatic software (version 5.0; TomoVision, Magog, Canada) was used to measure psoas muscle area (PMA), subcutaneous fat area (SFA), and visceral fat area (VFA). Detailed information on these methods and their clinical utility has been described in our recent studies [22, 23].

### 2.4 Definitions and Calculations of Variables

Euthyroid was defined as FT4 (11.0–25.0 pmol/L), FT3 (3.1–6.8 pmol/L), and TSH (0.27–4.20 μIU/mL) within the reference ranges. Severe obesity has been shown to be associated with increased serum TSH and FT3 levels in euthyroid adults [24]. Accordingly, in this study, patients with TSH levels above the reference range but with FT4 and FT3 not below the lower limit of normal, or with FT3 levels above the reference range but with TSH not below the lower limit of normal, were classified as having normal thyroid function. Central indices of thyroid hormone sensitivity were calculated using the following formulas: Thyrotropin index (TSHI) = ln(TSH (μIU/mL)) + 0.1345 × FT4 (pmol/L). Thyrotrophic thyroxine resistance index (TT4RI) = FT4 (pmol/L) × TSH (μIU/mL). For TSHI and TT4RI, higher values indicated lower central sensitivity to thyroid hormones. The peripheral index of thyroid hormone sensitivity was calculated as FT3/FT4 ratio = FT3 (pmol/L)/FT4 (pmol/L). Higher values of this ratio indicated higher peripheral sensitivity to thyroid hormones [25]. Percent total weight loss (%TWL) for this study was calculated as follows: 100  ×  (baseline weight − follow‐up weight)/baseline weight. Successful WL was defined as a TWL equal to or higher than 25%. DR was defined as a fasting glucose  < 5.6 mmol/L (100 mg/dL) and haemoglobin A1c (HbA1c) level  <  6.0% (42 mmol/mol) without antidiabetic medications, whereas long‐term DR was defined as remission lasting for at least 3 years after surgery. The primary outcomes were WL and DR during the 3 year follow‐up period after bariatric surgery. Diabetes, hypertension, and dyslipidemia were defined as in previous studies [26].

### 2.5 Statistical Analysis

Data normality was assessed using the Kolmogorov–Smirnov test. Normally distributed data, skewed data, and categorical variables were presented as mean (standard deviation), medians (interquartile range), and percentages, respectively. Group differences were analysed using the Chi‐squared or Fisher's exact test for categorical variables, and independent‐sample *t*‐test and Mann–Whitney *U* test for continuous factors. The paired *t*‐test and Wilcoxon signed‐rank test were conducted for follow‐up comparisons.

The correlation between thyroid parameters and clinical indicators was calculated using Spearman's rank coefficient and visualised by heatmap in Python (version 3.6.3) with Matplotlib (version 2.1.1). Breakpoint analysis was conducted using the ‘segmented, ggplot2, Hmisc, and rms’ packages in RStudio. The inflection point is one that minimises the sum residual square error of the two linear segments, above and below this point. It should be noted that this breakpoint does not represent a definitive biological threshold but rather a statistical representation of the data, aiding in understanding the segmented relationship between the variables. Participants in the longitudinal study were stratified into sex‐specific tertiles based on their baseline FT3 levels, with the lowest tertile serving as the reference group. Binary logistic regression models were used to estimate odds ratio (OR) and 95% confidence interval (CI) for successful WL and DR, while linear regression models were used to examine the independent association between baseline FT3 tertiles and the magnitude of WL following bariatric surgery. To minimise potential residual confounding, covariates in multivariable‐adjusted models with progressive adjustment were selected based on their well‐established or probable association with WL or DR after bariatric surgery. The Cox proportional hazards model was implemented using the ‘rms’ package in RStudio to analyse time‐to‐event hazard ratios (HR), incorporating restricted cubic splines (RCS) to examine and visualise the dose‐response relationship between FT3 (as a continuous variable) and successful WL and DR. The follow‐up period was precise to the day, with the primary analysis focussing on event outcomes within 1 year after bariatric surgery. Data censoring occurred at the study's conclusion. We validated the proportional hazard assumption using the Schoenfeld residual test. Kaplan–Meier curves and log‐rank tests were evaluated using the ‘survival’ package in RStudio. The cut points of baseline FT3 identified in the RCS analysis at a HR of 1.0 were stratified by sex to avoid the influence of significant sex‐related differences in serum FT3 levels.

A *p* < 0.05 (two‐tailed tests) was considered statistically significant. Because missing data were less than 5%, listwise deletion was used. All analyses were performed using SPSS version 25.0 (SPSS Inc., Chicago, IL, USA) and RStudio software v.1.3.959 (https://www.r‐project.org/; R, Vienna, Austria).

## 3 Results

### 3.1 Clinical Characteristics of the Participants in the Cross‐Sectional Study

A total of 1567 patients were enroled (Supporting Information S1: Table S1). Males did not differ significantly in age compared with females but exhibited higher BMI, waistline, blood glucose levels, and triglycerides. Body composition showed significant sex differences, with males having significantly higher PMA, VFA and FFMI (*p* < 0.001), while females showed greater SFA, BFP, and FMI (*p* ≤ 0.001). Significant differences were also observed in serum thyroid hormone profiles between sexes, with FT3 exhibiting the greatest difference, followed by TSH and FT4. Furthermore, males had significantly higher FT3/FT4 ratios but lower TSHI and TT4RI compared to females (*p* < 0.001).

### 3.2 Correlations Between Thyroid Parameters and Obesity‐Related Indices

**FIGURE 1:**
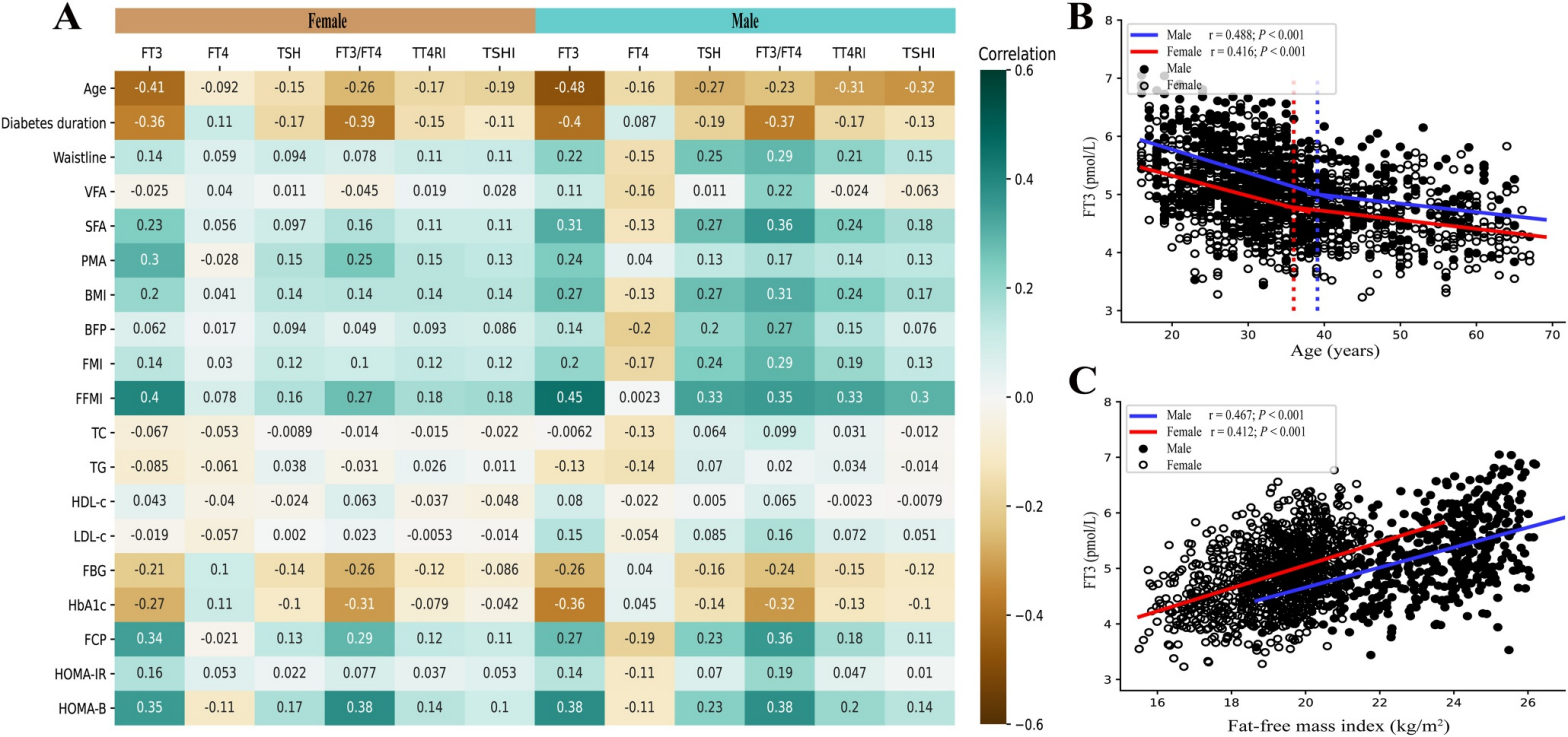
Relationship between thyroid parameters and obesity‐related indices in the cross‐sectional study population (1039 females and 528 males). (A) Spearman correlations between thyroid parameters and clinical features. The heat map visualisation shows the Spearman correlation coefficients for clinical characteristics. The colour bar indicates the Spearman's *r* value for each pair of variables, with darker colours representing stronger correlations. The analysis indicated that the correlations between serum FT3 and age, as well as between FT3 and FFMI, were the strongest. (B–C) The best‐fit relationships of serum FT3 with age and FFMI. (B) Serum FT3 and age. (C) Serum FT3 in relation to FFMI. Each circle represents a single participant in the study. The breakpoints, showing a sharp change in slope, are indicated by a dashed line in the corresponding colour. Segmental linear regression is applied if the correlation is significantly better than that of linear regression (*p* < 0.05). Pearson correlation coefficients and associated *p*‐values are shown for the female and male populations in the regression model. Breakpoints of serum FT3 were 4.773 pmol/L (females) and 5.001 pmol/L (males) for age.

Figure 1 showed that serum FT3 exhibited stronger correlations with obesity and metabolic indicators regardless of sex compared with other thyroid parameters. Among the variables, age and FFMI showed the strongest correlations with FT3. Furthermore, we employed segmented regression analysis to demonstrate the best‐fit relationships. Surprisingly, a nonlinear relationship existed between FT3 and age, while FT3 showed a linear correlation with FFMI. FT3 decreased until reaching the breakpoint (FT3 = 5.001 for males and 4.773 for females), after which it flattened (*p* < 0.01). The comparison of linear regression and segmented linear regression for the best‐fit relationship is presented in Supporting Information S1: Table S2.

### 3.3 Baseline and Postoperative Characteristics of Bariatric Surgery Cohort

In all, 1034 patients from the cross‐sectional population were included in the longitudinal study (Table 1). Significant improvements in serum lipid profiles, glycaemic profiles, and metabolic diseases were observed after weight loss (all *p* < 0.001). Both FT3 and TSH levels decreased significantly after surgery regardless of sex (*p* < 0.001), with FT3 showing the greatest reduction. FT4 changes differed by sex, with a significant decrease in females (*p* < 0.001) but no change in males (*p* = 0.286). Additionally, the FT3/FT4 ratio, TSHI, and TT4RI all decreased significantly in both sexes after bariatric surgery (*p* < 0.001).

**TABLE 1:** Clinical characteristics of the study population before and 1 year after bariatric surgery.

Variable	Total (*n* = 1034)			Female (*n* = 731)				Male (*n* = 303)			
	Baseline	1 year	*p* value	Baseline	1 year	*t/Z* value	*p* value	Baseline	1 year	*t/Z* value	*p* value
Age, years	36.4 ± 11.7			36.1 ± 11.5				37.1 ± 12.3			
Sleeve/RYGB, *n*	813/221			609/122				204/99			
BMI, kg/m^2^	36.3 ± 6.0	26.1 ± 4.2	< 0.001	36.1 ± 5.8	25.8 ± 4.1	76.9	< 0.001	36.8 ± 6.6	26.9 ± 4.3***	41.2	< 0.001
Waistline, cm	112.8 ± 13.9	90.1 ± 11.8	< 0.001	110.5 ± 12.6	88.5 ± 11.2	56.5	< 0.001	117.5 ± 15.2***	93.6 ± 12.3***	37.1	< 0.001
Thyroid parameters											
FT3, pmol/L	5.0 ± 0.72	4.2 ± 0.64	< 0.001	4.9 ± 0.70	4.1 ± 0.56	24.5	< 0.001	5.2 ± 0.72***	4.6 ± 0.65***	12.1	< 0.001
FT4, pmol/L	16.4 ± 2.2	16.1 ± 2.2	< 0.001	16.3 ± 2.2	15.9 ± 2.1	4.2	< 0.001	16.8 ± 2.3***	16.6 ± 2.3***	1.1	0.286
TSH, μIU/mL	2.4 (1.7, 3.5)	1.9 (1.2, 2.7)	< 0.001	2.6 (1.8, 3.7)	2.0 (1.3, 2.8)	−10.9	< 0.001	2.1(1.5, 2.9)***	1.6(1.1, 2.3)***	−6.9	< 0.001
FT3/FT4 ratios	0.31 ± 0.05	0.27 ± 0.04	< 0.001	0.30 ± 0.05	0.26 ± 0.04	18.9	< 0.001	0.31 ± 0.06*	0.28 ± 0.04***	9.2	< 0.001
TSHI	3.1 ± 0.70	2.7 ± 0.69	< 0.001	3.1 ± 0.71	2.7 ± 0.70	10.6	< 0.001	3.0 ± 0.66**	2.7 ± 0.65	6.9	< 0.001
TT4RI	39.0 (27.1, 56.4)	29.6 (18.7, 43.2)	< 0.001	42.8 (29.6, 59.1)	31.0 (19.0, 45.3)	−11.3	< 0.001	33.6 (23.6, 48.1)***	27.0 (17.7, 39.2)**	−7.0	< 0.001
Lipid profiles											
TC, mmol/L	5.1 ± 1.2	4.7 ± 1.1	< 0.001	5.2 ± 1.2	4.8 ± 1.1	6.9	< 0.001	5.0 ± 1.2	4.5 ± 1.2***	6.6	< 0.001
TG, mmol/L	1.7 (1.3, 2.5)	0.89 (0.68, 1.2)	< 0.001	1.6 (1.2, 2.2)	0.87 (0.66, 1.1)	−20.0	< 0.001	2.2 (1.4, 3.2)***	0.97(0.71, 1.4)***	−14.1	< 0.001
HDL‐c, mmol/L	1.1 ± 0.27	1.3 ± 0.33	< 0.001	1.1 ± 0.26	1.4 ± 0.33	−20.9	< 0.001	0.97 ± 0.26***	1.2 ± 0.28***	−11.6	< 0.001
LDL‐c, mmol/L	3.1 ± 0.84	2.8 ± 0.89	< 0.001	3.2 ± 0.83	2.8 ± 0.84	10.8	< 0.001	2.9 ± 0.85***	2.7 ± 0.99	3.5	0.001
Glycaemic profiles											
FPG, mmol/L	7.2 ± 2.8	5.0 ± 1.1	< 0.001	7.0 ± 2.8	4.9 ± 1.0	20.0	< 0.001	7.6 ± 2.8**	5.2 ± 1.2***	15.6	< 0.001
2‐h PBG, mmol/L	10.6 ± 4.4	5.9 ± 2.5	< 0.001	10.1 ± 4.2	5.6 ± 2.1	24.7	< 0.001	11.8 ± 4.7***	6.6 ± 3.0***	16.5	< 0.001
FCP, ng/mL	3.9 ± 1.8	2.1 ± 0.74	< 0.001	3.8 ± 1.7	2.0 ± 0.67	27.6	< 0.001	4.1 ± 1.9*	2.3 ± 0.84***	17.4	< 0.001
HbA1c, %	7.1 ± 1.9	5.6 ± 0.78	< 0.001	6.9 ± 1.8	5.5 ± 0.73	21.1	< 0.001	7.5 ± 2.0***	5.7 ± 0.87*	17.0	< 0.001
HOMA‐IR	7.3 (4.6, 11.8)	1.4 (0.93, 2.2)	< 0.001	6.9 (4.3, 11.6)	1.4 (0.90, 2.1)	−21.1	< 0.001	8.4(5.4, 12.1)***	1.7 (1.0, 2.5)***	−14.3	< 0.001
HOMA‐B	201.2 (92.0, 357.9)	107.5 (63.9, 172.1)	< 0.001	205.5 (104.0.358.5)	108.3 (67.1, 170.9)	−13.5	< 0.001	181.1 (73.6, 351.8)	106.3 (56.9, 175.7)	−7.8	< 0.001
Body composition											
BFP, %	43.4 ± 8.3	31.4 ± 6.8	< 0.001	46.2 ± 6.9	34.1 ± 5.5	75.4	< 0.001	36.4 ± 7.2***	24.9 ± 5.1***	40.4	< 0.001
FFMI, kg/m^2^	20.2 ± 2.3	17.8 ± 2.2	< 0.001	19.0 ± 1.2	16.8 ± 1.5	51.4	< 0.001	22.9 ± 1.8***	20.1 ± 2.0***	40.8	< 0.001
FMI, kg/m^2^	16.1 ± 5.5	8.4 ± 2.9	< 0.001	17.1 ± 5.3	9.0 ± 2.9	60.3	< 0.001	13.8 ± 5.3***	6.9 ± 2.5***	31.7	< 0.001
No. of patients^a^	369			238				131			
PMA, cm^2^	26.2 ± 8.4	22.0 ± 7.4	< 0.001	21.4 ± 4.1	17.6 ± 3.6	27.2	< 0.001	34.9 ± 6.9***	29.9 ± 5.8***	17.5	< 0.001
VFA, cm^2^	167.7 ± 59.0	61.5 ± 32.9	< 0.001	159.7 ± 50.8	60.2 ± 28.3	36.5	< 0.001	182.2 ± 69.4***	63.9 ± 39.9	25.1	< 0.001
SFA, cm^2^	368.6 ± 145.6	215.7 ± 102.3	< 0.001	391.1 ± 139.6	231.9 ± 97.8	28.0	< 0.001	327.9 ± 148.0***	186.3 ± 104.1***	18.5	< 0.001
Diabetes, *n* (%)	557 (53.9)	113 (10.9)	< 0.001	349 (47.7)	61 (8.3)		< 0.001	208 (68.6)	52 (17.2)		< 0.001
OHA, *n* (%)	341 (33.0)	35 (3.4)	< 0.001	203 (27.8)	25 (3.4)		< 0.001	138 (45.5)	10 (3.3)		< 0.001
Insulin therapy, *n* (%)	156 (15.1)	8 (0.8)	< 0.001	85 (11.6)	2 (0.3)		< 0.001	71 (23.4)	6 (2.0)		< 0.001
Hypertension, *n* (%)	563 (54.4)	188 (18.2)	< 0.001	349 (47.7)	109 (14.9)		< 0.001	214 (70.6)	79 (26.1)		< 0.001
Dyslipidemia, *n* (%)	711 (68.8)	256 (24.8)	< 0.001	457 (62.5)	150 (20.5)		< 0.001	254 (83.8)	106 (35.0)		< 0.001

*Note:* Data are presented as mean ± SD, median (IQR), or *n* (%).

Abbreviations: 2‐h PBG, 2‐h plasma glucose; BFP, body fat percentage; BMI, body mass index; CCBs, calcium channel blockers; FCP, fasting C‐peptide; FFMI, fat‐free mass index; FMI, fat mass index. FPG, fasting plasma glucose; FT3, free triiodothyronine; FT4, free thyroxine; HbA1c, haemoglobin A1c; HDL‐c, high‐density lipoprotein cholesterol; HOMA‐B, HOMA of β*‐c*ell function; HOMA‐IR, homoeostasis model assessment of insulin resistance; LDL‐c, low‐density lipoprotein cholesterol; OHA, oral hypoglycemic agents; PMA, psoas muscle area; RAASi, renin‐angiotensin‐aldosterone system inhibitors; RYGB, Roux‐en‐Y gastric bypass; SFA, subcutaneous fat area; TC, total cholesterol; TG, triglycerides cholesterol; TSH, thyroid‐stimulating hormone; TSHI, TSH index; TT4RI, thyrotrophic T4 resistance index; VFA, visceral fat area.

^a^ Number of patients who underwent abdominal MRI examination before and 1 year after bariatric surgery.

*p < 0.05, **p < 0.01, ***p < 0.001, compared with female patients at the corresponding time point.

### 3.4 Association Between Baseline Serum FT3 and Weight Loss After Bariatric Surgery

As shown in Table 2, significant differences were observed in the percent and absolute BMI decreases 1 year after bariatric surgery according to baseline FT3 tertiles, regardless of sex (*p* for trend < 0.001). The highest FT3 tertile was significantly associated with greater weight reduction in both males and females in model 3. However, after additional adjustment for baseline glycaemic parameters, this association remained highly significant in females (*p* for trend < 0.01) but disappeared in males. The associations between baseline FT3 tertiles and successful WL are presented in Supporting Information S1: Table S3. Consistent with the results in Table 2, high baseline FT3 was significantly associated with successful WL 1 year after bariatric surgery (*p* < 0.001), and this association was more pronounced in females.

**TABLE 2:** Changes in body weight 1 year after bariatric surgery according to tertiles of baseline serum FT3 levels.

	Mean changes (SD)	Additional changes after bariatric surgery (B, 95% CI)				
		Unadjusted model	Model 1	Model 2	Model 3	Model 4
Females (*n* = 731)						
Percent BMI, %						
Tertile 1 (< 4.58 pmol/L)	25.6 ± 7.0	Reference	Reference	Reference	Reference	Reference
Tertile 2 (4.58–5.09 pmol/L)	28.7 ± 7.2	3.1 (1.8–4.4)***	3.2 (1.9–4.5)***	1.9 (0.77–3.1)**	1.8 (0.64–3.0)**	1.5 (0.29–2.6)*
Tertile 3 (> 5.09 pmol/L)	30.0 ± 7.1	4.4 (3.1–5.6)***	4.5 (3.2–5.8)***	2.7 (1.4–3.9)***	2.5 (1.3–3.8)***	2.0 (0.74–3.3)**
*p* for trend		< 0.001	< 0.001	< 0.001	< 0.001	0.002
Absolute BMI, kg/m^2^						
Tertile 1 (< 4.58 pmol/L)	9.1 ± 3.4	Reference	Reference	Reference	Reference	Reference
Tertile 2 (4.58–5.09 pmol/L)	10.5 ± 3.7	1.4 (0.76–2.0)***	1.4 (0.77–2.0)***	0.73 (0.27–1.2)**	0.71 (0.25–1.2)**	0.59 (0.12–1.1)*
Tertile 3 (> 5.09 pmol/L)	11.2 ± 3.5	2.1 (1.4–2.7)***	2.0 (1.4–2.7)***	0.98 (0.49–1.5)***	0.95 (0.47–1.4)***	0.75 (0.25–1.3)**
*p* for trend		< 0.001	< 0.001	< 0.001	< 0.001	0.004
Males (*n* = 303)						
Percent BMI, %						
Tertile 1 (< 4.86 pmol/L)	23.2 ± 7.5	Reference	Reference	Reference	Reference	Reference
Tertile 2 (4.86–5.42 pmol/L)	26.3 ± 7.8	3.1 (1.0–5.2)**	3.3 (1.2–5.4)**	1.9 (−0.03–3.8)	1.9 (0.02–3.8)*	1.3 (−0.53–3.2)
Tertile 3 (> 5.42 pmol/L)	28.5 ± 7.5	5.2 (3.1–7.3)***	5.4 (3.3–7.5)***	2.4 (0.36–4.5)*	2.3 (0.23–4.3)*	1.4 (−0.69–3.4)
*p* for trend		< 0.001	< 0.001	0.025	0.034	0.211
Absolute BMI, kg/m^2^						
Tertile 1 (< 4.86 pmol/L)	8.4 ± 3.9	Reference	Reference	Reference	Reference	Reference
Tertile 2 (4.86–5.42 pmol/L)	9.9 ± 4.1	1.5 (0.39–2.6)**	1.6 (0.53–2.8)**	0.67 (−0.06–1.4)	0.69 (−0.38–1.4)	0.47 (−0.25–1.2)
Tertile 3 (> 5.42 pmol/L)	11.2 ± 4.0	2.8 (1.6–3.9)***	2.9 (1.7–4.0)***	0.81 (0.02–1.6)*	0.78 (−0.01–1.6)	0.47 (−0.32–1.3)
*p* for trend		< 0.001	< 0.001	0.049	0.059	0.264

*Note:* The test for trend was based on the median value of baseline serum FT3 for each tertile. Model 1 was adjusted for preoperative thyroid function (TSH and FT4 in the model of FT3 tertiles). Model 2 was adjusted for covariates in Model 1 plus age, baseline BMI, type of surgery. Model 3 was adjusted for covariates in Model 2 plus waistline, BFP, FFMI, and FMI at baseline. Model 4 was adjusted for covariates in Model 3 plus the presence or absence of diabetes at baseline, fasting C‐peptide, HbA1c, and HOMA‐IR at baseline.

Abbreviations: B, unstandardised coefficients; CI, confidence interval; SD, standard deviation.

*p < 0.05, **p < 0.01, ***p < 0.001, compared with the reference category.

### 3.5 Preoperative Serum FT3 Is Associated With Diabetes Remission at 1 Year After Bariatric Surgery

Significantly higher FT3 levels were observed in the DR group at baseline and even 1 year after surgery compared with the non‐DR group (Supporting Information S1: Table S4). As shown in Table 3, the probability of DR increased with higher FT3 tertiles, regardless of sex (*p* for trend < 0.001). After adjusting for preoperative thyroid function and serum lipid profiles at baseline, the adjusted OR for females in the highest tertile was 7.42 (*p* < 0.001), while for males it was 2.96 (*p* = 0.008), compared with the lowest tertile. In model 5, each unit increase in baseline FT3 level was associated with a 1.81‐fold increase in the probability of DR in females (OR 2.81 [1.53–5.18], *p* = 0.001), whereas this association was not observed in males.

**TABLE 3:** Odds ratio and 95% CI for diabetes remission 1 year after bariatric surgery by tertiles of baseline serum FT3 levels among patients with diabetes.

	Serum FT3 levels in females (*n* = 349)			*p* for trend	Per‐unit FT3 increase
	Tertile 1 (< 4.36 pmol/L)	Tertile 2 (4.36–4.91 pmol/L)	Tertile 3 (> 4.91 pmol/L)		
Median, pmol/L	4.11	4.67	5.37		
No. of patients	117	119	113		
No. of diabetes remission (%)	55 (47.0)	79 (66.4)	97 (85.8)		
Unadjusted	1.00	2.23 (1.32–3.77)**	6.83 (3.60–13.0)***	< 0.001	3.88 (2.48–6.09)***
Model 1	1.00	2.24 (1.32–3.81)**	7.04 (3.61–13.7)***	< 0.001	4.15 (2.58–6.67)***
Model 2	1.00	2.45 (1.41–4.27)**	7.42 (3.75–14.7)***	< 0.001	4.38 (2.67–7.21)***
Model 3	1.00	2.25 (1.26–4.02)**	5.53 (2.71–11.3)***	< 0.001	3.51 (2.09–5.90)***
Model 4	1.00	2.16 (1.20–3.90)*	5.94 (2.86–12.3)***	< 0.001	3.63 (2.14–6.15)***
Model 5	1.00	2.10 (1.06–4.18)*	4.01 (1.74–9.24)**	0.001	2.81 (1.53–5.18)**

	Serum FT3 levels in males (*n* = 208)			*p* for trend	Per‐unit FT3 increase
	Tertile 1 (< 4.71 pmol/L)	Tertile 2 (4.71–5.28 pmol/L)	Tertile 3 (> 5.28 pmol/L)		
Median, pmol/L	4.33	4.99	5.62		
No. of patients	70	70	68		
No. of diabetes remission (%)	30 (42.9)	43 (61.4)	50 (73.5)		
Unadjusted	1.00	2.12 (1.08–4.17)*	3.70 (1.81–7.59)***	< 0.001	2.07 (1.33–3.21)**
Model 1	1.00	2.21 (1.11–4.43)*	3.80 (1.78–8.10)***	0.001	2.15 (1.34–3.44)**
Model 2	1.00	1.72 (0.82–3.62)	2.96 (1.33–6.59)**	0.008	1.80 (1.12–2.88)*
Model 3	1.00	1.16 (0.48–2.76)	1.80 (0.69–4.70)	0.237	1.34 (0.78–2.32)
Model 4	1.00	1.17 (0.48–2.81)	1.79 (0.68–4.70)	0.245	1.36 (0.79–2.36)
Model 5	1.00	0.79 (0.27–2.34)	1.02 (0.30–3.48)	0.964	1.02 (0.52–1.99)

*Note:* The test for trend was based on the median value of baseline serum FT3 for each tertile. Model 1 was adjusted for preoperative thyroid function (TSH and FT4). Model 2 was adjusted for the variables in model 1 plus TC, TG, HDL‐c, and LDL‐c at baseline. Model 3 was adjusted for the variables in model 2 plus age, baseline BMI, type of surgery. Model 4 was adjusted for the variables in model 3 plus waistline, BFP, FFMI, and FMI at baseline. Model 5 was adjusted for the variables in model 4 plus diabetes duration, insulin usage, fasting C‐peptide, HbA1c, and HOMA‐IR at baseline.

Abbreviations: CI, confidence interval; No., number.

*p < 0.05, **p < 0.01, ***p < 0.001, compared with the first tertile or per 1 pmol/L increase in FT3.

### 3.6 The Dose‐Response Relationship of Baseline Serum FT3 Levels With Successful Weight Loss and Diabetes Remission

**FIGURE 2:**
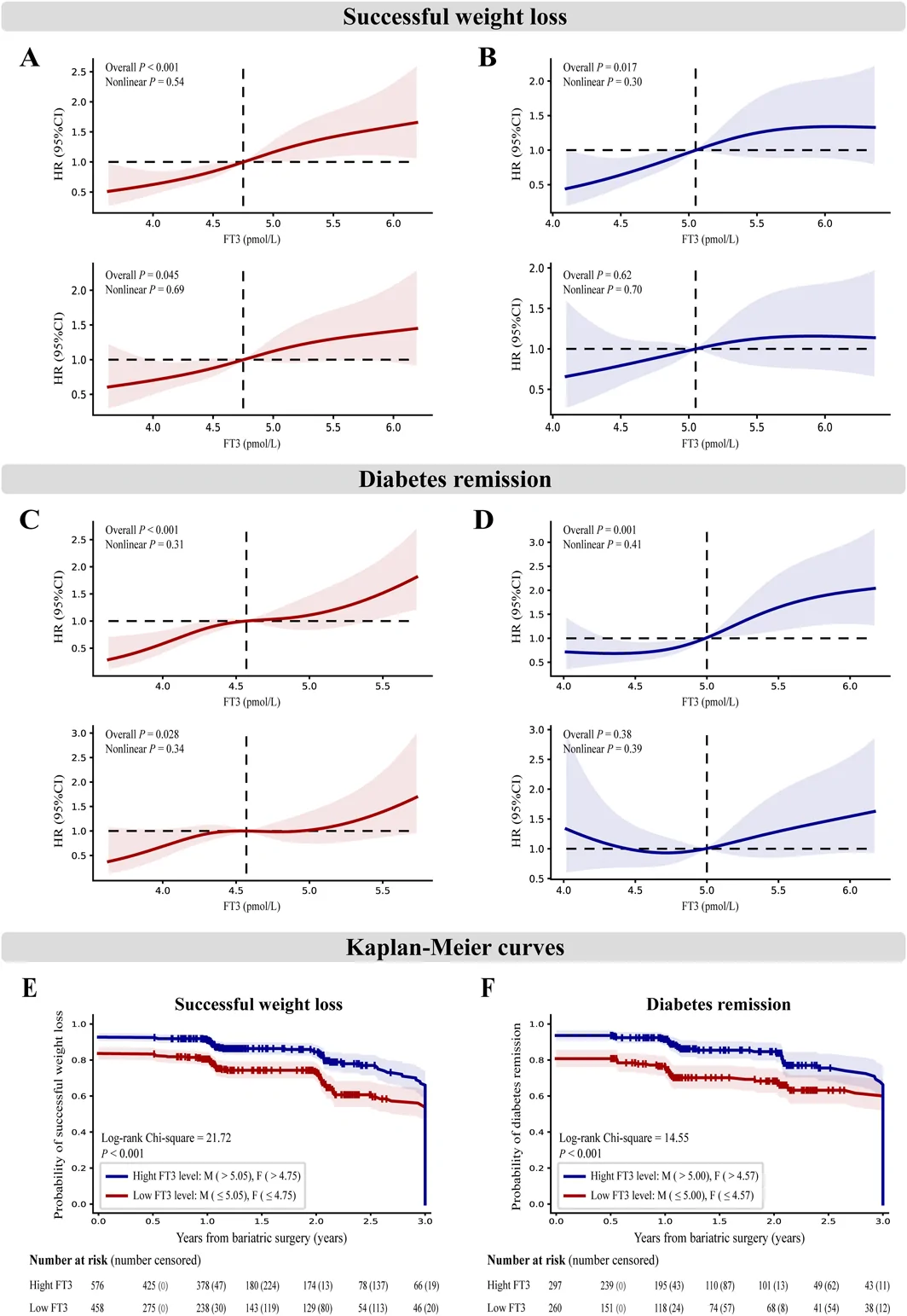
Analysis of the associations of baseline serum FT3 levels with successful weight loss and diabetes remission. (A–D) Restricted cubic splines for successful weight loss and diabetes remission 1 year after bariatric surgery among females and males. Four knots were set at the 5th, 35th, 65th and 95th percentiles. The red line and shading represent the HR and its 95% CI for females, while the blue line represents the same for males. A vertical dotted line indicates the threshold value of baseline serum FT3, while a horizontal dotted line represents an HR of 1.0. For Panels (A) and (B), the upper half of each panel shows the model adjusted for preoperative thyroid function (TSH and FT4), while the lower half is further adjusted for age, baseline BMI, type of surgery, and presence/absence of diabetes at baseline. For Panels (C) and (D), the upper half of each panel represents the model adjusted for preoperative thyroid function (TSH and FT4), and the lower half is further adjusted for age, baseline BMI, type of surgery, diabetes duration, insulin usage, fasting C‐peptide, HbA1c, HOMA‐IR at baseline. (E–F) Kaplan–Meier plots of long‐term successful weight loss and diabetes remission during the 3 year follow‐up. Follow‐up ranged from 0.5 to 3.0 years for both successful weight loss (mean [SD] follow‐up: 2.0 [0.9] years; median [IQR] follow‐up: 2.0 [1.0–3.0] years), and diabetes remission (mean [SD] follow‐up: 2.0 [0.9] years; median [IQR] follow‐up: 2.1 [1.1–3.0] years). The Kaplan–Meier plots were based on the sex‐specific threshold value of baseline serum FT3 identified in the RCS analysis. The number at risk indicated the number of patients who achieved successful weight loss or diabetes remission at each time point after bariatric surgery. F, female; M, male.

As shown in Figure 2, due to the uneven distribution of sample sizes and increased uncertainty in model estimation at the margins, we observed that the confidence intervals of all RCS curves widened even with slight deviations near HR = 1. Furthermore, the *p* values for the tests of linearity hypotheses in all models were > 0.05, confirming a linear relationship of baseline FT3 levels with WL and DR. As baseline FT3 levels increased, the probability of achieving successful WL and DR significantly increased in the thyroid function‐adjusted model, regardless of sex (*p*‐overall < 0.05). However, these dose‐response relationships disappeared in males after further adjustments. The optimal FT3 thresholds were 4.75 for females and 5.05 for males in achieving successful WL, and 4.57 for females and 5.00 for males in DR. When preoperative FT3 levels were below these thresholds, the risk of failing to achieve ideal glycaemic control and weight loss significantly increased. We stratified the entire cohort according to the FT3 threshold identified in the RCS analysis, and constructed Kaplan‐Meier survival curves. Compared with patients with higher baseline FT3 levels, those with lower FT3 levels had significantly lower probabilities of both successful WL and DR during the 3 year follow‐up (log‐rank *p* < 0.001).

## 4 Discussion

In this study, we found that a nonlinear relationship existed between FT3 and age, whereas FT3 showed a linear correlation with FFMI. In cohort analysis, high baseline FT3 levels (rather than TSH and FT4) were significantly associated with successful WL and DR at 1 and 3 years after bariatric surgery, exhibiting a dose‐dependent relationship. Notably, the pattern of these associations was similar in females and males, but stronger in females. To our knowledge, these findings have not been reported in the literature.

Obesity was associated with elevated serum levels of TSH and FT3, but not with FT4 levels [24]. Furthermore, obesity was linked to increased peripheral sensitivity and decreased central sensitivity to thyroid hormones. FT3 is the active form of thyroid hormone, involved in stimulating REEs [10, 27]. Therefore, it can be inferred that changes in thyroid hormone sensitivity associated with obesity aim to elevate FT3 levels, thereby increasing energy expenditure and limiting weight gain. This helps to explain why FT3 shows the strongest associations with adiposity and metabolic parameters compared with other thyroid indicators. Previous studies have emphasised the role of age and fat‐free mass in determining levothyroxine requirements in adults with hypothyroidism [28, 29]. Similarly, FT3 showed the strongest correlation with age and FFMI in this study. In fact, serum FT3 was shown to negatively correlate with age [30]. However, our study first demonstrates that FT3 exhibits a breakpoint effect with age independent of sex. A recent study suggested that as TSH levels increase, FT3/FT4 ratios increase until age 40, but this increase does not occur in older age groups [31]. Therefore, the breakpoint effect of FT3 with age is likely related to the cliff‐like decline in TSH action or deiodinase activity with age. Given that human skeletal muscle is the major site of type II iodothyronine deiodinase activity and thermogenesis [32], the parallel increase between FT3 and FFMI is understandable. Furthermore, the underlying mechanisms of elevated TSH and FT3 levels in individuals with obesity remain unclear [33]. Unlike the hypothesis that adipose tissue expansion mediates increased TSH secretion [34], our study found that muscle mass, particularly FFMI, was significantly positively correlated with both FT3 and TSH levels. This suggests that the elevation of TSH and FT3 in individuals with obesity may be related to increased muscle mass.

Consistent with our findings, most studies have shown that bariatric surgery is associated with a decrease in TSH and FT3 levels [35]. However, this change is probably mediated by weight loss rather than an intrinsic effect of bariatric surgery [36]. Similar phenomena have been observed in patients with obesity after non‐surgical weight loss [27, 37]. On the other hand, the impact of bariatric surgery on FT4 levels is more controversial [34, 38, 39]. In our study, FT4 significantly decreased in females but showed no significant change in males after surgery. Given the increased thyroxine requirement, obesity could increase the risk of hypothyroidism [17]. Interestingly, the associations between obesity and hypothyroidism also show sex differences, with obesity significantly increasing the risk of hypothyroidism in females but not in males [40]. Taken together, our results suggest that females may have a more severe thyroid burden before surgery and experience greater improvements in thyroid function after bariatric surgery, highlighting significant sex differences in thyroid health and outcomes from bariatric surgery. Furthermore, consistent with existing literature [27, 41], the elevated peripheral and decreased central sensitivity to thyroid hormones in individuals with obesity normalised following bariatric surgery. FT3 plays a central role in the adaptive changes in thyroid hormone sensitivity, likely explaining why it undergoes the most significant changes after surgery compared to other thyroid indicators.

Obesity is associated with increased FT3 levels, which decrease after bariatric surgery [13, 35, 37]. High preoperative FT3 increases energy expenditure and limits weight gain, while low postoperative FT3 enhances energy conservation and promotes weight regain. This indicates the presence of a specific body weight set‐point, above which FT3 levels increase to promote weight loss, and below which FT3 levels decrease to restore weight. The ‘physiological’ FT3 signal helps maintain weight balance. From this perspective, the higher the pre‐operative FT3, the greater the potential to reach the body weight set‐point after surgery. Since improvements in total body insulin sensitivity are weight‐dependent and WL is a strong predictor of DR after bariatric surgery [15, 19], it follows that the association between baseline FT3 and DR is likely a result of its positive effect on weight reduction. Given that the difference in FT3 levels before surgery is more pronounced, baseline FT3 significantly impacts postoperative WL and DR.

Preoperative FT3 was associated with short‐ and long‐term WL and DR after bariatric surgery. Although previous studies have suggested that preoperative FT3 levels may be associated with the extent of excess weight loss after surgery [42], our data further demonstrate that sex has a significant influence on these associations. At lower serum FT3 levels in females, both WL and DR increased with rising FT3 levels. In contrast, the associations with WL and DR were weaker at higher serum FT3 levels in males, suggesting a threshold effect. As baseline FT3 increases, both WL and DR tend to reach a plateau. In the FT3 versus WL and DR plots, females predominantly occupy the linear part of the curve on the left, while males occupy the flat part on the right. Ultimately, the substantial difference in serum FT3 levels between males and females may be a critical factor explaining the sex differences in the association of baseline FT3 with WL and DR. Of note, our hypothesis aligns with a recent important study, which found a significant association between lean body mass and cardiovascular parameters in females but not in males [43]. Our findings further validate this sex‐difference pattern in the field of obesity and indicate that sex‐specific effects arising from differences in body composition and metabolic levels may be more widespread. Together with the FT3 inflection points identified in the cross‐sectional study, our exploratory analysis suggested FT3 levels ≤ 5.0 pmol/L for males and ≤ 4.57 pmol/L for females as potential thresholds associated with suboptimal outcomes. Because hypothyroidism is approximately tenfold more prevalent in females than in males [44], these cut‐points, which require external validation, may be particularly relevant to females. Furthermore, individuals with low preoperative FT3 levels may warrant intensified postoperative monitoring. However, given the observational nature of this study, whether preoperative thyroid function optimization (e.g., thyroid hormone supplementation) could improve postoperative outcomes remains speculative and requires evaluation in prospective interventional studies.

Our study had several limitations. First, this was an observational cohort study, and the associations identified cannot be interpreted as causal. Secondly, REEs were not directly measured. Because REE is central to the proposed mechanism linking FT3 to postoperative weight loss, this limitation precluded direct verification of the metabolic pathway linking preoperative FT3 to observed surgical outcomes. Finally, although the participants with thyroid disease were excluded, serum thyroid antibody levels were not routinely checked. Nonetheless, given the large sample size, this limitation is unlikely to affect the interpretation of our results.

In conclusion, serum FT3 shows a nonlinear age‐related breakpoint and a linear correlation with FFMI in euthyroid adults with obesity. Low preoperative FT3 at or below sex‐specific exploratory thresholds (≤ 5.0 pmol/L in males and ≤ 4.57 pmol/L in females) was associated with suboptimal short‐ and long‐term WL and DR after bariatric surgery, particularly in females. These thresholds may support preoperative risk stratification and inform individualised treatment selection as obesity therapies expand, including bariatric surgery and GLP‐1 receptor agonists. Future randomized controlled trials should confirm these thresholds and determine whether any strategy targeting patients with low preoperative FT3 can improve surgical outcomes.

## Author Contributions

S.L., Y.T., Y.M., H.Y., and F.L. conceived and designed the study. Y.T. and S.L. contributed to data acquisition. S.L. and H.Y. performed the literature search and statistical analysis. S.L. wrote the manuscript. Y.B., J.Y., H.Y., and F.L. supervised the study and critically revised the manuscript for important intellectual content. H.Z. contributed to standardized surgical procedures. Y.X. contributed to the standardized abdominal MRI examination. All authors contributed to the article and approved the submitted version.

## Funding

This work was supported by the National Natural Science Foundation of China (82601612, 82470881), the Noncommunicable Chronic Diseases‐National Science and Technology Major Project (2023ZD0509205), the Shanghai Municipal Health Commission of General Programme (202440117), and the Shanghai Municipal Education Commission—Gaofeng Clinical Medicine Grant Support (20172025).

## Conflicts of Interest

The authors declare no conflicts of interest.

## Supporting information


Supporting Information S1

## Data Availability

The data that support the findings of this study are available on request from the corresponding author. The data are not publicly available due to privacy or ethical restrictions.

## References

[1] P. Saeedi , I. Petersohn , P. Salpea , et al., “Global and Regional Diabetes Prevalence Estimates for 2019 and Projections for 2030 and 2045: Results From the International Diabetes Federation Diabetes Atlas, 9(th) Edition,” Diabetes Research and Clinical Practice 157 (2019): 107843, 10.1016/j.diabres.2019.107843. 31518657

[2] A. Afshin , M. H. Forouzanfar , M. B. Reitsma , et al., “Health Effects of Overweight and Obesity in 195 Countries Over 25 Years,” New England Journal of Medicine 377, no. 1 (2017): 13–27, 10.1056/NEJMoa1614362. 28604169 PMC5477817

[3] G. Mingrone , S. Panunzi , A. De Gaetano , et al., “Metabolic Surgery Versus Conventional Medical Therapy in Patients With Type 2 Diabetes: 10‐Year Follow‐up of an Open‐Label, Single‐Centre, Randomised Controlled Trial,” Lancet 397, no. 10271 (2021): 293–304, 10.1016/s0140-6736(20)32649-0. 33485454

[4] G. Scozzari , R. Passera , R. Benvenga , M. Toppino , and M. Morino , “Age as a Long‐Term Prognostic Factor in Bariatric Surgery,” Annals of Surgery 256, no. 5 (2012): 724–728: Discussion 728–729, 10.1097/sla.0b013e3182734113. 23095615

[5] N. Kanerva , I. Larsson , M. Peltonen , A. K. Lindroos , and L. M. Carlsson , “Changes in Total Energy Intake and Macronutrient Composition After Bariatric Surgery Predict Long‐Term Weight Outcome: Findings From the Swedish Obese Subjects (SOS) Study,” American Journal of Clinical Nutrition 106, no. 1 (2017): 136–145, 10.3945/ajcn.116.149112. 28515062 PMC5486198

[6] K. M. McTigue , R. Wellman , E. Nauman , et al., “Comparing the 5‐Year Diabetes Outcomes of Sleeve Gastrectomy and Gastric Bypass: The National Patient‐Centered Clinical Research Network (Pcornet) Bariatric Study,” JAMA Surgery 155, no. 5 (2020): e200087, 10.1001/jamasurg.2020.0087. 32129809 PMC7057171

[7] J. Debédat , N. Sokolovska , M. Coupaye , et al., “Long‐Term Relapse of Type 2 Diabetes After Roux‐en‐Y Gastric Bypass: Prediction and Clinical Relevance,” Diabetes Care 41, no. 10 (2018): 2086–2095, 10.2337/dc18-0567. 30082327

[8] A. C. Bianco and E. A. McAninch , “The Role of Thyroid Hormone and Brown Adipose Tissue in Energy Homoeostasis,” Lancet Diabetes and Endocrinology 1, no. 3 (2013): 250–258, 10.1016/s2213-8587(13)70069-x. 24622373 PMC4976626

[9] K. D. Hall , “Modeling Metabolic Adaptations and Energy Regulation in Humans,” Annual Review of Nutrition 32, no. 1 (2012): 35–54, 10.1146/annurev-nutr-071811-150705. 22540251

[10] R. Mullur , Y. Y. Liu , and G. A. Brent , “Thyroid Hormone Regulation of Metabolism,” Physiological Reviews 94, no. 2 (2014): 355–382, 10.1152/physrev.00030.2013. 24692351 PMC4044302

[11] N. Knudsen , P. Laurberg , L. B. Rasmussen , et al., “Small Differences in Thyroid Function May be Important for Body Mass Index and the Occurrence of Obesity in the Population,” Journal of Clinical Endocrinology and Metabolism 90, no. 7 (2005): 4019–4024, 10.1210/jc.2004-2225. 15870128

[12] T. Reinehr , “Obesity and Thyroid Function,” Molecular and Cellular Endocrinology 316, no. 2 (2010): 165–171, 10.1016/j.mce.2009.06.005. 19540303

[13] G. Karavani , D. Strich , S. Edri , and D. Gillis , “Increases in Thyrotropin Within the Near‐Normal Range are Associated With Increased Triiodothyronine but Not Increased Thyroxine in the Pediatric Age Group,” Journal of Clinical Endocrinology and Metabolism 99, no. 8 (2014): E1471–E1475, 10.1210/jc.2014-1441. 24878053

[14] K. Sjöholm , P. Pajunen , P. Jacobson , et al., “Incidence and Remission of Type 2 Diabetes in Relation to Degree of Obesity at Baseline and 2 Year Weight Change: The Swedish Obese Subjects (SOS) Study,” Diabetologia 58, no. 7 (2015): 1448–1453, 10.1007/s00125-015-3591-y. 25924987

[15] B. Halpern and M. C. Mancini , “Metabolic Surgery for the Treatment of Type 2 Diabetes in Patients With BMI Lower Than 35 kg/m(2) : Why Caution is Still Needed,” Obesity Reviews 20, no. 5 (2019): 633–647, 10.1111/obr.12837. 30821085

[16] F. Rong , H. Dai , Y. Wu , et al., “Association Between Thyroid Dysfunction and Type 2 Diabetes: A Meta‐Analysis of Prospective Observational Studies,” BMC Medicine 19, no. 1 (2021): 257, 10.1186/s12916-021-02121-2. 34670571 PMC8529738

[17] B. Biondi , G. J. Kahaly , and R. P. Robertson , “Thyroid Dysfunction and Diabetes Mellitus: Two Closely Associated Disorders,” Endocrine Reviews 40, no. 3 (2019): 789–824, 10.1210/er.2018-00163. 30649221 PMC6507635

[18] G. Dimitriadis , P. Mitrou , V. Lambadiari , et al., “Insulin Action in Adipose Tissue and Muscle in Hypothyroidism,” Journal of Clinical Endocrinology and Metabolism 91, no. 12 (2006): 4930–4937, 10.1210/jc.2006-0478. 17003097

[19] S. Madsbad , C. Dirksen , and J. J. Holst , “Mechanisms of Changes in Glucose Metabolism and Bodyweight After Bariatric Surgery,” Lancet Diabetes and Endocrinology 2, no. 2 (2014): 152–164, 10.1016/s2213-8587(13)70218-3. 24622719

[20] N. Gronich , S. N. Deftereos , I. Lavi , A. S. Persidis , D. R. Abernethy , and G. Rennert , “Hypothyroidism is a Risk Factor for New‐Onset Diabetes: A Cohort Study,” Diabetes Care 38, no. 9 (2015): 1657–1664, 10.2337/dc14-2515. 26070591

[21] Y. Du , J. Zhang , G. Chen , and Z. Sun , “Formulation and Interpretation of the Chinese Guidelines for Surgical Treatment of Obesity and Type 2 Diabetes Mellitus,” Bioscience Trends 15, no. 5 (2021): 299–304, 10.5582/bst.2021.01287. 34334581

[22] S. Li , P. Zhang , J. Di , et al., “Associations of Change in Body Fat Percentage With Baseline Body Composition and Diabetes Remission After Bariatric Surgery,” Obesity 32, no. 5 (2024): 871–887, 10.1002/oby.24003. 38515375

[23] S. Li , H. Yu , P. Zhang , et al., “The Nonlinear Relationship Between Psoas Cross‐Sectional Area and BMI: A New Observation and Its Insights Into Diabetes Remission After Roux‐en‐Y Gastric Bypass,” Diabetes Care 44, no. 12 (2021): 2783–2786, 10.2337/dc20-2907. 34645667 PMC8669530

[24] P. N. Taylor , R. Richmond , N. Davies , et al., “Paradoxical Relationship Between Body Mass Index and Thyroid Hormone Levels: A Study Using Mendelian Randomization,” Journal of Clinical Endocrinology and Metabolism 101, no. 2 (2016): 730–738, 10.1210/jc.2015-3505. 26595101 PMC4880123

[25] H. Sun , W. Zhu , J. Liu , Y. An , Y. Wang , and G. Wang , “Reduced Sensitivity to Thyroid Hormones is Associated With High Remnant Cholesterol Levels in Chinese Euthyroid Adults,” Journal of Clinical Endocrinology and Metabolism 108, no. 1 (2022): 166–174, 10.1210/clinem/dgac523. 36071542

[26] Y. Si , H. Zhang , X. Han , et al., “Percentage of Maximum Weight Lost as an Optimal Parameter of Weight Regain After Bariatric Surgery in Chinese Patients With Diabetes,” Obesity 31, no. 6 (2023): 1538–1546, 10.1002/oby.23764. 37133427

[27] R. V. Agnihothri , A. B. Courville , J. D. Linderman , et al., “Moderate Weight Loss is Sufficient to Affect Thyroid Hormone Homeostasis and Inhibit its Peripheral Conversion,” Thyroid 24, no. 1 (2014): 19–26, 10.1089/thy.2013.0055. 23902316 PMC3887425

[28] J. J. Cunningham and U. S. Barzel , “Lean Body Mass is a Predictor of the Daily Requirement for Thyroid Hormone in Older Men and Women,” Journal of the American Geriatrics Society 32, no. 3 (1984): 204–207, 10.1111/j.1532-5415.1984.tb02003.x. 6699335

[29] C. Mele , M. A. Tagliaferri , L. Pagano , et al., “Levothyroxine Replacement in Obese Adults: The Role of Metabolic Variables and Aging on Thyroid Testing Abnormalities,” Journal of Clinical Endocrinology and Metabolism 104, no. 12 (2019): 6265–6274, 10.1210/jc.2019-00773. 31265068

[30] A. Corsonello , A. Montesanto , M. Berardelli , et al., “A Cross‐Section Analysis of FT3 Age‐Related Changes in a Group of Old and Oldest‐Old Subjects, Including Centenarians’ Relatives, Shows That a Down‐Regulated Thyroid Function Has a Familial Component and is Related to Longevity,” Age and Ageing 39, no. 6 (2010): 723–727, 10.1093/ageing/afq116. 20843963 PMC2956534

[31] D. Strich , G. Karavani , S. Edri , and D. Gillis , “TSH Enhancement of FT4 to FT3 Conversion is Age Dependent,” European Journal of Endocrinology 175, no. 1 (2016): 49–54, 10.1530/eje-16-0007. 27150496

[32] P. R. Larsen , “Type 2 Iodothyronine Deiodinase in Human Skeletal Muscle: New Insights Into Its Physiological Role and Regulation,” Journal of Clinical Endocrinology and Metabolism 94, no. 6 (2009): 1893–1895, 10.1210/jc.2009-0791. 19494166 PMC2690423

[33] M. Blüher , A. Rudich , N. Klöting , et al., “Two Patterns of Adipokine and Other Biomarker Dynamics in a Long‐Term Weight Loss Intervention,” Diabetes Care 35, no. 2 (2012): 342–349, 10.2337/dc11-1267. 22190676 PMC3263919

[34] J. Knezevic , C. Starchl , A. Tmava Berisha , and K. Amrein , “Thyroid‐Gut‐Axis: How Does the Microbiota Influence Thyroid Function?,” Nutrients 12, no. 6 (2020): 1769, 10.3390/nu12061769. 32545596 PMC7353203

[35] N. F. Butte , M. L. Brandt , W. W. Wong , et al., “Energetic Adaptations Persist After Bariatric Surgery in Severely Obese Adolescents,” Obesity 23, no. 3 (2015): 591–601, 10.1002/oby.20994. 25707380 PMC4340087

[36] Z. Tian , Y. Nie , Z. Li , et al., “Total Weight Loss Induces the Alteration in Thyroid Function After Bariatric Surgery,” Frontiers in Endocrinology 15 (2024): 1333033, 10.3389/fendo.2024.1333033. 38352711 PMC10861714

[37] B. Wolters , N. Lass , and T. Reinehr , “TSH and Free Triiodothyronine Concentrations are Associated With Weight Loss in a Lifestyle Intervention and Weight Regain Afterwards in Obese Children,” European Journal of Endocrinology 168, no. 3 (2013): 323–329, 10.1530/eje-12-0981. 23211576

[38] J. S. Neves , S. Castro Oliveira , P. Souteiro , et al., “Effect of Weight Loss After Bariatric Surgery on Thyroid‐Stimulating Hormone Levels in Patients With Morbid Obesity and Normal Thyroid Function,” Obesity Surgery 28, no. 1 (2018): 97–103, 10.1007/s11695-017-2792-5. 28725979

[39] B. Guan , Y. Chen , J. Yang , W. Yang , and C. Wang , “Effect of Bariatric Surgery on Thyroid Function in Obese Patients: A Systematic Review and Meta‐Analysis,” Obesity Surgery 27, no. 12 (2017): 3292–3305, 10.1007/s11695-017-2965-2. 29039052

[40] B. Wang , R. Song , W. He , et al., “Sex Differences in the Associations of Obesity With Hypothyroidism and Thyroid Autoimmunity Among Chinese Adults,” Frontiers in Physiology 9 (2018): 1397, 10.3389/fphys.2018.01397. 30337885 PMC6180185

[41] P. Juiz‐Valiña , M. Cordido , E. Outeiriño‐Blanco , et al., “Central Resistance to Thyroid Hormones in Morbidly Obese Subjects is Reversed After Bariatric Surgery‐Induced Weight Loss,” Journal of Clinical Medicine 9, no. 2 (2020): 359, 10.3390/jcm9020359. 32012985 PMC7073690

[42] J. S. Neves , P. Souteiro , S. C. Oliveira , et al., “Preoperative Thyroid Function and Weight Loss After Bariatric Surgery,” International Journal of Obesity 43, no. 2 (2019): 432–436, 10.1038/s41366-018-0071-8. 29769703

[43] C. Diaz‐Canestro , B. Pentz , A. Sehgal , R. Yang , A. Xu , and D. Montero , “Lean Body Mass and the Cardiovascular System Constitute a Female‐Specific Relationship,” Science Translational Medicine 14, no. 667 (2022): eabo2641, 10.1126/scitranslmed.abo2641. 36260693

[44] P. N. Taylor , D. Albrecht , A. Scholz , et al., “Global Epidemiology of Hyperthyroidism and Hypothyroidism,” Nature Reviews Endocrinology 14, no. 5 (2018): 301–316, 10.1038/nrendo.2018.18. 29569622

