# Supplementary material for: Low Preoperative Free Triiodothyronine, Particularly in Females, Is Associated With Reduced Weight Loss and Diabetes Remission After Bariatric Surgery in Euthyroid Adults
Source: Diabetes Metab Res Rev. 2026 Sep 14;42(6):e70228. doi: 10.1002/dmrr.70228 (PMC13572958; doi:10.1002/dmrr.70228)
Supplement: Supplementary file 1 [file DMRR-42-e70228-s001.docx]

**Supplementary Information**

**Article title**

Low preoperative free triiodothyronine, particularly in females, is associated with reduced weight loss and diabetes remission after bariatric surgery in euthyroid adults

**Author names**

Shaobo Li PhD ^1,2,†^, Yinfang Tu PhD ^1,†^, Hongwei Zhang PhD ^3^, Yunfeng Xiao MD ^4^, Yuqian Bao PhD ^1^, Jun Yin PhD ^1^, Yujin Ma MD ^2^, Haoyong Yu PhD ^1,*^ and Fengjing Liu PhD ^1,*^

**Authors’ affiliations**

^1^ Department of Endocrinology and Metabolism, Shanghai Sixth People’s Hospital Affiliated to Shanghai Jiao Tong University School of Medicine, Shanghai Clinical Center for Diabetes, Shanghai Key Clinical Center for Metabolic Disease, Shanghai Diabetes Institute, Shanghai Key Laboratory of Diabetes Mellitus, Shanghai 200233, China

^2^ Henan Key Laboratory of Rare Diseases, Endocrinology and Metabolism Center, The First Affiliated Hospital, and College of Clinical Medicine of Henan University of Science and Technology, Luoyang 471003, China

^3^ Department of General Surgery, Shanghai Sixth People’s Hospital Affiliated to Shanghai Jiao Tong University School of Medicine, Shanghai 200233, China

^4^ Department of Radiology, Shanghai Sixth People’s Hospital Affiliated to Shanghai Jiao Tong University School of Medicine, Shanghai 200233, China

Shaobo Li and Yinfang Tu contributed equally to this work.

**Affiliation and e-mail address of the corresponding author**

Prof. Yujin Ma ([mayujin126@126.com](mailto:mayujin126@126.com)), Prof. Haoyong Yu ([yuhaoyong@shsmu.edu.cn](mailto:yuhaoyong@shsmu.edu.cn)), and Prof. Fengjing Liu (liufengjing@shsmu.edu.cn).

**Tables**

**Table S1.** Characteristics of cross-sectional study participants stratified by sex............................................ 3

**Table S2.** Comparison of linear regression with segmented linear regression used for the best-fit relationship of serum FT3 levels with age and FFMI in males and females....................................................... 5

**Table S3.** Relative risks and 95% CI for successful weight loss one year after bariatric surgery by tertiles of baseline serum FT3 levels ............................................................................................................... 6

**Table S4.** Characteristics of the diabetes remission and non-remission groups before and 1 year after bariatric surgery............................................................................................................................. 7

**Figures**

**Figure S1.** The flow chart of the study population ........................................................................................ 9

**Table S1** Characteristics of cross-sectional study participants stratified by sex.

| Variables | Total (*n* = 1567) | Female (*n* = 1039) | Male (*n* = 528) | *t/t'/Z* value | *p* value |
| --- | --- | --- | --- | --- | --- |
| Age, years | 35.4 ± 11.4 | 35.1 ± 11.3 | 36.1 ± 11.6 | -1.57 | 0.116 |
| BMI, kg/m^2^ | 36.9 ± 6.4 | 36.6 ± 6.1 | 37.4 ± 6.9 | -2.36 | 0.019 |
| Waistline, cm | 114.4 ± 14.9 | 111.8 ± 13.6 | 119.4 ± 15.9 | -9.46 | <0.001 |
| SBP, mmHg | 133.4 ± 16.4 | 130.8 ± 15.8 | 138.4 ± 16.4 | -8.83 | <0.001 |
| DBP, mmHg | 85.8 ± 11.7 | 84.6 ± 11.3 | 88.3 ± 12.1 | -5.98 | <0.001 |
| Scr, µmol/L | 60.4 ± 15.5 | 54.2 ± 11.1 | 72.3 ± 15.9 | -23.3 | <0.001 |
| eGFR, mL/min/1.73 m^2^ | 114.4 ± 24.3 | 115.9 ± 24.5 | 112.2 ± 23.8 | 2.00 | 0.046 |
| Lipid profiles |  |  |  |  |  |
| TC, mmol/L | 5.1 ± 1.1 | 5.1 ± 1.0 | 5.0 ± 1.1 | 2.40 | 0.016 |
| TG, mmol/L | 1.7 (1.2, 2.4) | 1.6 (1.2, 2.2) | 2.1 (1.4, 3.1) | -8.02 | <0.001 |
| HDL-c, mmol/L | 1.1 ± 0.26 | 1.1 ± 0.26 | 0.96 ± 0.23 | 12.2 | <0.001 |
| LDL-c, mmol/L | 3.1 ± 0.82 | 3.2 ± 0.83 | 3.0 ± 0.80 | 4.75 | <0.001 |
| Glycemic profiles |  |  |  |  |  |
| FPG, mmol/L | 7.0 ± 2.9 | 6.9 ± 2.8 | 7.4 ± 2.9 | -3.19 | 0.001 |
| 2-h PBG, mmol/L | 9.9 ± 4.5 | 9.5 ± 4.4 | 10.8 ± 4.7 | -5.27 | <0.001 |
| FCP, ng/mL | 4.1 ± 1.8 | 3.9 ± 1.7 | 4.4 ± 2.1 | -4.41 | <0.001 |
| HbA1c, % | 6.9 ± 1.9 | 6.7 ± 1.8 | 7.3 ± 1.9 | -5.01 | <0.001 |
| HbA1c, mmol/mol | 52.0 ± 20.8 | 50.0 ± 19.7 | 56.0 ± 20.8 | -5.01 | <0.001 |
| HOMA-IR | 7.3 (4.6, 11.7) | 6.9 (4.4, 11.2) | 8.5 (5.1, 13.0) | -4.41 | <0.001 |
| HOMA-B | 207.5 (100.6, 378.1) | 208.6 (111.0, 365.2) | 204.0 (83.1, 397.4) | -0.45 | 0.654 |
| Thyroid parameters |  |  |  |  |  |
| FT3, pmol/L | 5.0 ± 0.69 | 4.9 ± 0.63 | 5.3 ± 0.73 | -9.67 | <0.001 |
| FT4, pmol/L | 16.4 ± 2.1 | 16.2 ± 2.0 | 16.9 ± 2.3 | -6.24 | <0.001 |
| TSH, μIU/mL | 2.4 (1.7, 3.5) | 2.6 (1.8, 3.7) | 2.1 (1.5, 3.0) | -7.17 | <0.001 |
| FT3/FT4 ratios | 0.31 ± 0.05 | 0.31 ± 0.05 | 0.32 ± 0.05 | -3.18 | 0.002 |
| TSHI | 3.1 ± 0.59 | 3.1 ± 0.59 | 3.0 ± 0.58 | 3.67 | <0.001 |
| TT4RI | 39.2 (27.9, 56.1) | 41.7 (29.6, 59.2) | 34.7 (24.9, 50.9) | -5.86 | <0.001 |
| Body composition measurements | |  |  |  |  |
| BFP, % | 43.4 ± 8.6 | 46.6 ± 7.2 | 37.0 ± 7.5 | 24.9 | <0.001 |
| FFMI, kg/m^2^ | 20.5 ± 2.4 | 19.1 ± 1.2 | 23.1 ± 1.8 | -44.9 | <0.001 |
| FMI, kg/m^2^ | 16.4 ± 5.8 | 17.5 ± 5.7 | 14.3 ± 5.6 | 10.5 | <0.001 |
| No. of patients ^†^ | 1005 | 675 | 330 |  |  |
| PMA, cm^2^ | 26.7 ± 8.0 | 22.5 ± 4.5 | 35.4 ± 6.5 | -32.6 | <0.001 |
| VFA, cm^2^ | 174.0 ± 67.4 | 160.2 ± 58.4 | 202.4 ± 75.5 | -8.94 | <0.001 |
| SFA, cm^2^ | 426.8 ± 164.6 | 439.3 ± 154.5 | 401.1 ± 181.1 | 3.30 | 0.001 |
| Diabetes, *n* (%) | 835 (53.3) | 506 (48.7) | 329 (62.3) |  | <0.001 |
| Hypertension, *n* (%) | 822 (52.5) | 480 (46.2) | 342 (64.8) |  | <0.001 |
| Dyslipidemia, *n* (%) | 1049 (66.9) | 632 (60.8) | 417 (79.0) |  | <0.001 |

*Note*: Data are presented as mean ± standard deviation (SD), median (interquartile range, IQR), or *n* (%).

Abbreviations: SBP, systolic blood pressure; DBP, diastolic blood pressure; Scr, serum creatinine; eGFR, estimated glomerular filtration rate; TC, total cholesterol; TG, triglycerides cholesterol; HDL-c, high-density lipoprotein cholesterol; LDL-c, low-density lipoprotein cholesterol; FPG, fasting plasma glucose; 2-h PBG, 2-hour plasma glucose; FCP, fasting C-peptide; HbA1c, hemoglobin A1c; HOMA-IR, homeostasis model assessment of insulin resistance; HOMA-B, HOMA of β-cell function; FT3, free triiodothyronine; FT4, free thyroxine; TSH, thyroid-stimulating hormone; TSHI, TSH index; TT4RI, thyrotrophic T4 resistance index; BFP, body fat percentage; FFMI, fat-free mass index; FMI, fat mass index. PMA, psoas muscle area; VFA, visceral fat area; SFA, subcutaneous fat area.

^†^ Number of patients who underwent abdominal MRI examination before surgery.

**Table S2** Comparison of linear regression with segmented linear regression used for the best-fit relationship of serum FT3 levels with age and FFMI in males and females.

|  | Linear regression | | Segmented linear regression | | | comparison | |
| --- | --- | --- | --- | --- | --- | --- | --- |
|  | **Slope** | **R^2^** | **Slope1** | **Slope2** | **R^2^** | **Breakpoint** | *p*-value |
| Male (*n* = 528) |  |  |  |  |  |  |  |
| Age | -0.0283 | 0.2199 | -0.0402 | -0.0146 | 0.2346 | (39.11, 5.001) | 0.0089 |
| Fat-free mass index | 0.1802 | 0.2187 | 0.1195 | 0.2426 | 0.2257 |  | 0.1064 |
| Female (*n* = 1039) |  |  |  |  |  |  |  |
| Age | -0.0222 | 0.1615 | -0.0342 | -0.0149 | 0.1713 | (36, 4.773) | 0.0029 |
| Fat-free mass index | 0.2070 | 0.1702 | 0.1892 | 0.3463 | 0.173 |  | 0.1844 |

Abbreviations: R^2^, coefficient of determination; Slope1, the slope before the breakpoint; Slope2, the slope after the breakpoint; *p*, *p*-value from the Log likelihood ratio test (linear regression model vs. segmented linear regression model).

**Table S3** Relative risks and 95% CI for successful weight loss one year after bariatric surgery by tertiles of baseline serum FT3 levels.

|  | **Serum FT3 levels in Females (*n* = 731)** | | | ***p* for trend** | **Per-unit FT3 increase** |
| --- | --- | --- | --- | --- | --- |
|  | **Tertile 1 (< 4.58 pmol/L)** | **Tertile 2 (4.58–5.09 pmol/L)** | **Tertile 3 (> 5.09 pmol/L)** |  |  |
| Median, pmol/L | 4.25 | 4.85 | 5.51 |  |  |
| No. of patients | 244 | 242 | 245 |  |  |
| No. of successful weight loss (%) | 131 (53.7) | 171 (70.7) | 187 (76.3) |  |  |
| Unadjusted | 1.00 | 2.11 (1.45–3.07) ^***^ | 2.78 (1.89–4.10) ^***^ | <0.001 | 1.79 (1.39–2.30) ^***^ |
| Model 1 | 1.00 | 2.20 (1.50–3.21) ^***^ | 2.99 (2.00–4.47) ^***^ | <0.001 | 1.94 (1.50–2.53) ^***^ |
| Model 2 | 1.00 | 2.19 (1.48–3.22) ^***^ | 2.88 (1.91–4.33) ^***^ | <0.001 | 1.95 (1.49–2.55) ^***^ |
| Model 3 | 1.00 | 1.77 (1.18–2.67) ^**^ | 2.03 (1.30–3.18) ^**^ | 0.002 | 1.49 (1.11–2.00) ^**^ |
| Model 4 | 1.00 | 1.74 (1.15–2.64) ^**^ | 2.00 (1.28–3.14) ^**^ | 0.002 | 1.47 (1.10–1.98) ^*^ |
| Model 5 | 1.00 | 1.53 (1.00–2.34) ^*^ | 1.66 (1.04–2.67) ^*^ | 0.035 | 1.29 (0.94–1.76) |
|  | **Serum FT3 levels in Males (*n* = 303)** | | | ***p* for trend** | **Per-unit FT3 increase** |
|  | **Tertile 1 (< 4.86 pmol/L)** | **Tertile 2 (4.86–5.42 pmol/L)** | **Tertile 3 (> 5.42 pmol/L)** |  |  |
| Median, pmol/L | 4.49 | 5.11 | 5.84 |  |  |
| No. of patients | 102 | 100 | 101 |  |  |
| No. of successful weight loss (%) | 38 (37.3) | 58 (58.0) | 68 (67.3) |  |  |
| Unadjusted | 1.00 | 2.33 (1.32–4.10) ^**^ | 3.47 (1.95–6.19) ^***^ | <0.001 | 2.19 (1.54–3.11) ^***^ |
| Model 1 | 1.00 | 2.47 (1.39–4.40) ^**^ | 3.68 (2.03–6.67) ^***^ | <0.001 | 2.31 (1.60–3.32) ^***^ |
| Model 2 | 1.00 | 2.30 (1.25–4.24) ^**^ | 3.40 (1.81–6.37) ^***^ | <0.001 | 2.17 (1.48–3.18) ^***^ |
| Model 3 | 1.00 | 2.11 (1.07–4.16) ^*^ | 2.25 (1.09–4.65) ^*^ | 0.036 | 1.62 (1.06–2.48) ^*^ |
| Model 4 | 1.00 | 2.21 (1.11–4.40) ^*^ | 2.25 (1.08–4.69) ^*^ | 0.040 | 1.63 (1.06–2.51) ^*^ |
| Model 5 | 1.00 | 1.94 (0.93–4.02) | 1.75 (0.80–3.86) | 0.202 | 1.31 (0.85–2.03) |

*Note*: Note: The test for trend was based on the median value of baseline serum FT3 for each tertile. Model 1 was adjusted for preoperative thyroid function (TSH and FT4). Model 2 was adjusted for the variables in model 1 plus systolic blood pressure, diastolic blood pressure, serum creatinine, TC, TG, HDL-c, and LDL-c at baseline. Model 3 was adjusted for the variables in model 2 plus age, baseline BMI, type of surgery, follow-up time at one year after surgery. Model 4 was adjusted for the variables in model 3 plus waist circumference, BFP, FFMI, and FMI at baseline. Model 5 was adjusted for the variables in model 4 plus the presence or absence of diabetes at baseline, fasting C-peptide, FBG, HbA1c, and HOMA-IR at baseline. Abbreviations: CI, confidence interval; No., number; ^*^ *p* < 0.05, ^**^ *p* < 0.01, ^***^ *p* < 0.001, compared with the first tertile or per 1 pmol/L increase in FT3.

**Table S4** Characteristics of the diabetes remission and non-remission groups before and 1 year after bariatric surgery.

|  | Female (*n* = 349) | | | | Male (*n* = 208) | | | |
| --- | --- | --- | --- | --- | --- | --- | --- | --- |
|  | **Remission (*n* = 231)** | | **No Remission (*n* = 118)** | | **Remission (*n* = 123)** | | No Remission (*n* = 85) | |
| Variable | **Baseline** | **1 Year** | **Baseline** | **1 Year** | **Baseline** | **1 Year** | **Baseline** | 1 Year |
| Age, years | 38.2 ± 11.5 |  | 45.5 ± 12.9 ^‡^ |  | 36.5 ± 11.4 |  | 46.4 ± 11.0 ^‡^ |  |
| Diabetes duration, years | 0.50 (0.10, 3.0) |  | 6.0 (2.0, 10.0) ^‡^ |  | 1.0 (0.10, 4.0) |  | 8.0 (5.0, 11.5) ^‡^ |  |
| Sleeve/RYGB, *n* | 172/59 |  | 57/61 |  | 81/42 |  | 25/60 |  |
| Follow-up time, years | 1.04 (1.00, 1.10) |  | 1.04 (1.01, 1.08) |  | 1.03 (1.00, 1.08) |  | 1.05(1.01,1.13) ^*^ |  |
| BMI, kg/m^2^ | 35.9 ± 5.6 | 25.5 ± 4.1 ^¶^ | 33.7 ± 5.9 ^†^ | 26.2 ± 4.4 ^¶^ | 36.6 ± 5.8 | 26.6 ± 4.1 ^¶^ | 31.4 ± 3.9 ^‡^ | 25.3 ± 3.5 ^* ¶^ |
| Weight, kg | 97.0 ± 17.2 | 68.9 ± 12.4 ^¶^ | 88.7 ± 16.6 ^‡^ | 68.9 ± 12.6 ^¶^ | 113.3 ± 21.4 | 82.3 ± 15.3 ^¶^ | 95.0 ± 15.4 ^‡^ | 76.7 ± 13.4 ^† ¶^ |
| Waistline, cm | 111.7 ± 12.6 | 88.6 ± 12.2 ^¶^ | 108.3 ± 12.9 ^*^ | 90.4 ± 11.4 ^¶^ | 118.7 ± 14.2 | 93.8 ± 12.2 ^¶^ | 106.4 ± 10.3 ^‡^ | 89.5 ± 10.8 ^* ¶^ |
| SBP, mmHg | 134.2 ± 16.8 | 122.5 ± 16.8 ^¶^ | 132.9 ± 15.2 | 123.6 ± 16.6 ^¶^ | 138.6 ± 16.0 | 124.0 ± 14.1 ^¶^ | 132.3 ± 14.1 ^†^ | 125.3 ± 15.4 ^\|\|^ |
| DBP, mmHg | 85.4 ± 11.5 | 77.5 ± 11.2 ^¶^ | 83.8 ± 10.6 | 76.5 ± 9.6 ^¶^ | 88.8 ± 12.5 | 79.3 ± 10.4 ^¶^ | 84.7 ± 10.0 ^*^ | 78.6 ± 9.8 ^¶^ |
| Scr, µmol/L | 53.4 ± 12.1 | 55.7 ± 10.8 ^¶^ | 54.0 ± 16.5 | 56.2 ± 14.3 ^§^ | 72.4 ± 21.7 | 71.9 ± 15.6 | 76.9 ± 24.3 | 74.9 ± 20.5 |
| Thyroid parameters |  |  |  |  |  |  |  |  |
| FT3, pmol/L | 4.9 ± 0.66 | 4.1 ± 0.57 ^¶^ | 4.4 ± 0.54 ^‡^ | 4.0 ± 0.59 ^¶^ | 5.2 ± 0.79 | 4.6 ± 0.68 ^¶^ | 4.8 ± 0.61 ^‡^ | 4.4 ± 0.69 ^* ¶^ |
| FT4, pmol/L | 16.5 ± 2.3 | 15.7 ± 2.2 ^¶^ | 16.1 ± 1.9 | 15.4 ± 2.1 ^¶^ | 16.9 ± 2.3 | 16.5 ± 2.0 ^§^ | 16.8 ± 2.4 | 15.9 ± 2.1 ^\|\|^ |
| TSH, μIU/mL | 2.4 (1.7, 3.5) | 2.0 (1.3, 2.9) ^¶^ | 2.5 (1.7, 3.4) | 2.1 (1.4, 3.0) ^\|\|^ | 2.1 (1.5, 2.9) | 1.7 (1.1, 2.7) ^¶^ | 1.7 (1.4, 2.4) | 1.5 (1.0, 2.2) ^§^ |
| FT3/FT4 ratios | 0.30 ± 0.05 | 0.26 ± 0.04 ^¶^ | 0.28 ± 0.04 ^‡^ | 0.26 ± 0.05 ^\|\|^ | 0.31 ± 0.05 | 0.28 ± 0.04 ^¶^ | 0.29 ± 0.06 ^*^ | 0.28 ± 0.05 ^§^ |
| TSHI | 3.1 ± 0.64 | 2.7 ± 0.67 ^¶^ | 3.0 ± 0.67 | 2.7 ± 0.60 ^¶^ | 2.9 ± 0.74 | 2.7 ± 0.67 ^¶^ | 2.8 ± 0.55 | 2.5 ± 0.63 ^* ¶^ |
| TT4RI | 37.7 (26.4, 55.1) | 30.9 (19.8, 44.7) ^¶^ | 38.0 (27.4, 53.6) | 31.2 (18.5, 46.6) ^¶^ | 34.9 (24.1, 49.9) | 28.7 (18.1, 43.6) ^¶^ | 29.0 (22.8, 42.0) | 23.1 (16.5, 33.5) ^\|\|^ |
| Lipid profiles |  |  |  |  |  |  |  |  |
| TC, mmol/L | 5.3 ± 1.2 | 4.8 ± 1.0 ^¶^ | 5.2 ± 1.4 | 4.8 ± 1.2 ^\|\|^ | 5.0 ± 1.2 | 4.4 ± 1.3 ^¶^ | 5.0 ± 1.3 | 4.3 ± 0.99 ^¶^ |
| TG, mmol/L | 1.9 (1.4, 2.5) | 0.85 (0.67, 1.1) ^¶^ | 1.9 (1.4, 2.6) | 1.1 (0.84, 1.4) ^‡ ¶^ | 2.2 (1.6, 3.0) | 0.89 (0.72, 1.2) ^¶^ | 2.3 (1.4, 3.8) | 1.3 (0.88, 1.6) ^‡ ¶^ |
| HDL-c, mmol/L | 1.1 ± 0.26 | 1.4 ± 0.31 ^¶^ | 1.1 ± 0.23 | 1.3 ± 0.30 ^¶^ | 0.95 ± 0.21 | 1.2 ± 0.24 ^¶^ | 0.97 ± 0.34 | 1.1 ± 0.27 ^¶^ |
| LDL-c, mmol/L | 3.2 ± 0.86 | 2.8 ± 0.81 ^¶^ | 3.1 ± 0.83 | 2.8 ± 0.87 ^\|\|^ | 2.9 ± 0.85 | 2.7 ± 1.0 ^§^ | 2.7 ± 0.84 | 2.5 ± 0.77 ^§^ |
| Glycemic profiles |  |  |  |  |  |  |  |  |
| FPG, mmol/L | 8.3 ± 2.9 | 4.6 ± 0.44 ^¶^ | 9.0 ± 3.1 ^*^ | 6.3 ± 1.5 ^‡ ¶^ | 8.1 ± 2.7 | 4.7 ± 0.49 ^¶^ | 9.4 ± 2.7 ^†^ | 6.6 ± 1.2 ^‡ ¶^ |
| 2-h PBG, mmol/L | 11.6 ± 4.0 | 5.2 ± 1.4 ^¶^ | 13.2 ± 4.1 ^†^ | 7.9 ± 3.1 ^‡ ¶^ | 12.5 ± 4.5 | 5.4 ± 1.8 ^¶^ | 14.0 ± 4.0 ^*^ | 9.7 ± 3.1 ^‡ ¶^ |
| FCP, ng/mL | 4.3 ± 2.0 | 2.0 ± 0.68 ^¶^ | 2.9 ± 1.2 ^‡^ | 2.1 ± 0.72 ^¶^ | 4.5 ± 2.1 | 2.3 ± 0.79 ^¶^ | 2.9 ± 1.4 ^‡^ | 2.0 ± 0.74 ^† ¶^ |
| HbA1c, % | 7.6 ± 1.7 | 5.4 ± 0.34 ^¶^ | 8.6 ± 1.8 ^‡^ | 6.6 ± 0.95 ^‡ ¶^ | 7.9 ± 1.9 | 5.3 ± 0.32 ^¶^ | 8.7 ± 1.7 ^†^ | 6.6 ± 1.1 ^‡ ¶^ |
| HOMA-IR | 9.0 (5.4, 14.5) | 1.3 (0.94, 1.9) ^¶^ | 7.8 (4.7, 14.4) | 2.0 (1.2, 3.0) ^‡ ¶^ | 9.7 (5.9, 15.2) | 1.4 (0.92, 2.1) ^¶^ | 8.8 (5.1, 11.9) | 1.9 (1.1, 2.9) ^† ¶^ |
| HOMA-B | 128.7  (71.2, 244.5) | 118.5  (78.7, 190.3) | 92.1  (47.6, 195.6) ^†^ | 59.2  (32.8, 104.4) ^‡ ¶^ | 157.0  (78.9, 283.0) | 121.8  (79.7, 194.6) ^§^ | 74.3  (40.4, 137.7) ^‡^ | 46.5  (27.8, 73.3) ^‡ ¶^ |
| Body composition |  |  |  |  |  |  |  |  |
| BFP, % | 46.5 ± 6.5 | 34.3 ± 5.2 ^¶^ | 45.6 ± 7.4 | 36.7 ± 5.7 ^‡ ¶^ | 36.1 ± 7.0 | 24.3 ± 5.2 ^¶^ | 32.1 ± 4.7 ^‡^ | 25.1 ± 4.3 ^¶^ |
| FFMI, kg/m^2^ | 18.9 ± 1.2 | 16.6 ± 1.6 ^¶^ | 18.0 ± 1.3 ^‡^ | 16.4 ± 1.6 ^¶^ | 23.0 ± 1.5 | 19.9 ± 1.9 ^¶^ | 21.1 ± 1.4 ^‡^ | 18.8 ± 1.7 ^‡ ¶^ |
| FMI, kg/m^2^ | 17.0 ± 5.1 | 8.9 ± 2.8 ^¶^ | 15.8 ± 5.6 ^*^ | 9.8 ± 3.2 ^† ¶^ | 13.6 ± 4.8 | 6.6 ± 2.5 ^¶^ | 10.2 ± 2.9 ^‡^ | 6.5 ± 2.0 ^¶^ |
| No. of patients ^a^ | 186 | 102 | 111 | 74 | 103 | 64 | 78 | 57 |
| PMA, cm^2^ | 22.2 ± 4.4 | 17.8 ± 3.5 ^¶^ | 20.0 ± 4.4 ^‡^ | 16.2 ± 3.5 ^† ¶^ | 36.2 ± 6.7 | 30.8 ± 5.8 ^¶^ | 32.7 ± 6.3 ^‡^ | 28.4 ± 5.7 ^* ¶^ |
| VFA, cm^2^ | 173.2 ± 61.0 | 59.7 ± 26.8 ^¶^ | 159.6 ± 55.4 | 61.0 ± 30.8 ^¶^ | 205.9 ± 77.0 | 49.9  (26.8, 87.0) ^¶^ | 179.7 ± 68.2 ^*^ | 48.1  (36.0, 82.6) ^¶^ |
| SFA, cm^2^ | 419.8 ± 141.7 | 227.6 ± 91.5 ^¶^ | 359.0 ± 155.0 ^†^ | 204.2 ± 94.1 ^¶^ | 405.7 ± 164.0 | 181.4  (120.9, 238.1) ^¶^ | 257.1 ± 100.8 ^‡^ | 137.3  (107.8,177.8) ^† ¶^ |
| OHA, *n* (%) | 113 (48.9) | 0 (0.0) ^¶^ | 90 (76.3) ^‡^ | 25 (21.2) ^‡ ¶^ | 69 (56.1) | 0 (0.0) ^¶^ | 69 (81.2) ^‡^ | 10 (11.8) ^‡ ¶^ |
| Insulin therapy, *n* (%) | 33 (14.3) | 0 (0.0) ^¶^ | 52 (44.1) ^‡^ | 2 (1.7) ^¶^ | 26 (21.1) | 0 (0.0) ^¶^ | 45 (52.9) ^‡^ | 6 (7.1) ^† ¶^ |

*Note*: Data are mean ± SD, median (IQR), or *n* (%).

^*^*p* < 0.050 between same-gender subgroups; ^†^*p* < 0.010 between same-gender subgroups; ^‡^*p* < 0.001 between same-gender subgroups.

^§^*p* < 0.050 within the same-gender subgroup vs*.* baseline; ^||^*p* < 0.010 within the same-gender subgroup vs. baseline; ^¶^*p* < 0.001 within the same-gender subgroup vs*.* baseline.

^a^ Number of patients who underwent abdominal MRI examination before and 1 year after bariatric surgery.

**Figure S1** The flow chart of the study population.

557 (M:208; F:349) patients included in the cohort for

diabetes remission study

Completed 1.5-year follow-up, *n*=346 (M:133; F:213)

Completed 2.0-year follow-up, *n*=325 (M:125; F:200)

Completed 2.5-year follow-up, *n*=209 (M:77; F:132)

Completed 3.0-year follow-up, *n*=186 (M:71; F:115)

1034 (M:303, F:731) patients included in bariatric surgery cohort for weight loss study

Completed 1.5-year follow-up, *n*=614 (M:194, F:420)

Completed 2.0-year follow-up, *n*=581 (M:185, F:396)

Completed 2.5-year follow-up, *n*=331 (M:111; F:220)

Completed 3.0-year follow-up, *n*=292 (M:100; F:192)

1567 (M:528, F:1039) patients included in cross-sectional study

Diagnosed with T2DM, *n*=835

Completed baseline MRI examinations, *n*=1005

Excluded: *n*=243 (M:79, F:164)

Aged younger than 16 years, *n*=8

A BMI of more than 56 kg/m^2^, *n*=31

A FCP of less than 1.0 ng/ml, *n*=25

Overt hypo- or hyperthyroidism, history of thyroid disease

or medication, *n*=54

Missing baseline FT3, FT4, TSH or other vital data, *n*=108

No eligible according to exclusion criteria, *n*=17

Excluded: *n*=477 (M:95; F:382)

No diagnosis of T2DM, *n*=477

Excluded: *n*=533 (M:225, F:308)

Abandoned surgery treatment, *n*=47

Without complete 1-year follow-up, *n*=486

1810 (M:607, F:1203) patients assessed for eligibility from February 2011 to November 2022

BMI, body mass index; FCP, fasting C-peptide; FT3, free triiodothyronine; FT4, free thyroxine; TSH, thyroid-stimulating hormone; T2DM, type 2 diabetes mellitus; MRI, magnetic resonance imaging; M, male; F, female.
